# Effects of straw returning combined with earthworm addition on nitrification and ammonia oxidizers in paddy soil

**DOI:** 10.3389/fmicb.2022.1069554

**Published:** 2022-12-16

**Authors:** Xiangqian Chu, Naling Bai, Xianqing Zheng, Quanhua Wang, Xi Pan, Shuangxi Li, Juanqin Zhang, Haiyun Zhang, Wenjie He, Feng Zhong, Weiguang Lv, Hanlin Zhang

**Affiliations:** ^1^Shanghai Academy of Agricultural Sciences, Eco-environmental Protection Institute, Shanghai, China; ^2^Shanghai Engineering Research Center of Plant Germplasm Resources, College of Life Sciences, Shanghai Normal University, Shanghai, China; ^3^Shanghai Agricultural Academy of Sciences, Key Laboratory of Integrated Rice-Fish Farming Ecosystem, Ministry of Agriculture and Rural Affairs, Shanghai, China; ^4^Faculty of Resources and Environmental Science, Hubei University, Wuhan, China; ^5^Agricultural Environment and Farmland Conservation Experiment Station of Ministry Agriculture, Shanghai, China

**Keywords:** ammonia-oxidizing microorganisms, straw returning, earthworm addition, soil nitrification, potential nitrification rates

## Abstract

**Introduction:**

Soil ammonia oxidation, which acts as the first and rate-limiting step of nitrification, is driven by ammonia-oxidizing bacteria (AOB), ammonia-oxidizing archaea (AOA) and complete ammonia oxidizer (comammox, amoA gene of clade-A and clade-B). Straw returning, widely used ecological technology in China, is an effective measure for promoting straw decomposition and soil nutrient cycling when combined with earthworm addition. However, the effects of straw returning combined with earthworm addition on soil ammonia oxidizers remain poorly understood.

**Methods:**

A 2-year plot experiment was conducted with 5 treatments: no fertilizer (CK); regular fertilization (RT); straw returning (SR); earthworm addition (W); straw returning + earthworm addition (SRW). The AOA, AOB, comammox clade-A and clade-B community microbial diversities and structures were investigated by high-throughput sequencing.

**Results:**

The results showed that (1) compared to RT treatment, W, SR, and SRW treatments all significantly increased the richness of AOA and comammox clade-A and clade-B (*p* < 0.05), and the richness of AOB was only significantly promoted by SRW treatment (*p* < 0.05). However, only SRW had a higher comammox clade-B diversity index than RT. (2) The ammonia oxidizer community structures were altered by both straw returning and earthworm addition. Soil NH_4_^+^-N was the critical environmental driver for altering the ammonia oxidizer community structure. (3) Compared with RT treatment, the soil potential nitrification rate (PNR) of W and SRW treatments increased by 1.19 and 1.20 times, respectively. The PNR was significantly positively correlated with AOB abundance (path coefficient = 0.712, *p* < 0.05) and negatively correlated with clade-B abundance (path coefficient = −0.106, *p* < 0.05).

**Discussion:**

This study provides scientific support for the application of straw returning combined with earthworm addition to improve soil nitrification with respect to soil ammonia-oxidizing microorganisms.

## Highlights

- Straw returning combined with earthworm increased the richness of ammonia oxidizers.
- NH_4_^+^-N was a critical driver for altering the ammonia oxidizer community structure.
- Earthworm addition significantly increased soil PNR.
- Under the alkaline paddy soil, AOB dominated in soil nitrification.

## 1. Introduction

Soil microbes are an important component of soil ecosystems (Jia et al., 2020). They directly or indirectly change the soil structure and nutrients through their activities and play a crucial role in soil nitrogen cycling (Cai et al., 2021). Nitrification, essential for the soil nitrogen cycling, is completed by the oxidation of ammonia and nitrite (Beeckman et al., 2018; Liu et al., 2019). Ammonia-oxidizing bacteria (AOB) and ammonia-oxidizing archaea (AOA) drive the oxidation of ammonia, which is the rate-limiting stage of the nitrification process (Könneke et al., 2005). AOA are generally considered to be the most abundant ammonia oxidizers in soil. However, in 2015, complete ammonia oxidizer (comammox) was found in aquaculture systems (Daims et al., 2015; van Kessel et al., 2015). Since then, comammox has been found in various systems, such as agricultural soil, forest soil, freshwater habitat, and sewage treatment plants (Wang Y. et al., 2017; Shi et al., 2020). In a recent study, comammox was the most abundant ammonia-oxidizing microorganism in forest, grassland, and agricultural soils and made a momentous contribution to soil nitrification (Hu et al., 2021). Determining the diversity, composition and contributions to agricultural soil nitrification of AOA, AOB, and comammox is of great significance for understanding the nitrogen cycle and improving agricultural management.

Straw returning is a widely used ecological measure of carbon sequestration and soil fertility enhancement in agroecosystems (Yang et al., 2019). Straw can affect the soil microbial community directly by its own decomposition or indirectly by altering soil physicochemical properties. Studies have revealed that rice straw and fertilization significantly increase AOB abundance and diversity, but AOA are almost unresponsive, possibly due to their mixotrophic character (Zhang et al., 2019). However, Chen et al. (2021) has found that though maize straw had no discernible impact on AOB in alkaline soil (pH = 7.82), it boosted the amoA gene abundance of AOA. The effects of straw returning on the ammonia-oxidizing community might be influenced by soil fertility, crop varieties, and agricultural management.

Recently, straw returning is questioned for the reasons of lack of nitrogen in the seedling stage by slow decay and the aggravation of crop diseases by pathogenicity of straw (Li et al., 2018). Under straw returning conditions, earthworm can promote straw decomposition, refine the soil structure, and improve soil drainage and aeration (Li et al., 2022). In vermicomposting systems, earthworm significantly promoted the net nitrification rate and the amount of AOA and AOB (Huang et al., 2017). In Cd-contaminated soil, earthworm promoted potential ammonia oxidation (PAO) by increasing AOB abundance but had no significant effect on soil net nitrification (Xu et al., 2018). Few researches have reported the influences of straw returning combined with earthworm addition on the variation of ammonia-oxidizing microorganisms. The mechanism of how earthworm and straw regulate soil ammonia-oxidizing microorganisms and thus affect nitrification remains to be elucidated.

In this study, high-throughput sequencing analysis was used to assess the abundance and community composition alterations of AOA, AOB, and comammox (amoA gene of clade-A and clade-B) in soil based on a 2-year plot trial of straw returning coupled with earthworm addition. Under the effects of straw returning and earthworm addition, the aims of this research were (1) to estimate the abundance, diversity, and community composition changes of AOA, AOB, and comammox; (2) to explore the key environmental factors influencing the ammonia-oxidizing microbial community; (3) to determine the relative contribution of ammonia-oxidizing microorganisms to nitrification, providing a scientific basis for the improvement of *in-situ* straw utilization technology.

## 2. Materials and methods

### 2.1. Site description and soil-based experiment design

The 2-year experiment was conducted in the Sanxing Experimental Station of Shanghai Academy of Agricultural Sciences, Chongming, Shanghai (31°41′20″ N, 121°33′47″ E). The annual average temperature of this area was 15.3°C, and the mean annual precipitation was 1003.7 mm. The soil texture was sandy loam. At the beginning of the trial test, the soil physicochemical characteristics were as follows: pH, 8.30 (soil: water = 1:2.5); soil organic carbon (SOC), 15.21 g·kg^−1^; total nitrogen (TN), 0.94 g·kg^−1^; total phosphorus (TP), 1.57 g·kg^−1^.

The cropping regime was rice-fallow rotation. The rice variety was Huayou 14 (*Oryza sativa* L.), which was artificially transplanted in mid-June and harvested in early November every year. There were five treatments: control with no fertilizer (CK); regular fertilization treatment (RT); straw returning + regular fertilization treatment (SR); earthworm addition + regular fertilization treatment (W); straw returning + earthworm addition + regular fertilization treatment (SRW). The earthworm species was *Pheretima guillelmi*. Each treatment was performed in three replicates, and all replicates were set by a random block design. Each plot measured 240 m^2^ and was separated by an impervious membrane. The same pure amounts of nitrogen (N), phosphorous (P), and potassium (K) (225, 112.5, and 255 kg·ha^−1^, respectively), according to the convention in Chongming area, were used in the four fertilization treatments throughout the rice season. Urea, Ca(H_2_PO_4_)_2_, and K_2_SO_4_ were the fertilizers used in the experiment. In the SR and SRW treatments, all straw was cut into small pieces of 7 ~ 10 cm and directly returned to the field. The average N, P, and K contents in the rice straw were 0.33, 0.19, and 1.35%, respectively. In the W and SRW treatments, 375 kg·ha^−1^ earthworms were applied to the field. Pit was dug (1 × 1 × 0.3 m) in the center of the paddy field, and 25 kg/666.67 m^2^ earthworms were added before covering the soil. Earthworms of the same size were released into the field after the rice harvest and harvested before the next rice planting season. Trapping method was used to harvest earthworms raised in the field before next rice season.

### 2.2. Field sampling and laboratory analyses

Soil samples were collected in April 2020. Using the S-type sampling method, five soil samples (0–20 cm) were drawn at random and combined into a single sample. The soil samples were preserved in fresh-keeping bags at a low temperature and brought back to the lab, in which stones, miscellaneous sundries, and animal and plant remains were eliminated. One portion of the samples were kept at −80°C for DNA extraction and high-throughput sequencing, and the remaining samples were air-dried to ascertain the soil physicochemical characteristics.

Potentiometry was used to estimate the soil pH (the water to soil ratio was 2.5:1); dichromate oxidation method was used to determine SOC content; soil TN was determined by the Kjeldahl method; TP was determined by the sulfuric acid-perchloric acid digestion method; available N (AN) was determined by the NaCl extraction method; the sodium hydrogen carbonate solution-Mo-Sb antispectrophotometric technique was used to determine the amount of accessible P (AP); exchangeable NH_4_^+^-N was determined by the direct distillation method; exchangeable NO_3_^−^-N was determined by a UV spectrophotometer (Persee T6, China).

The potential nitrification rate (PNR) was determined according to ISO Guideline 15685 (2012). The ammonium oxidation rate (μg (NO_2_^−^-N) ·g^−1^·h^−1^ dry soil) was calculated between the different NO_2_^−^-N concentrations at different measuring times.

### 2.3. Soil DNA analyses

#### 2.3.1. DNA extraction

Soil DNA was extracted using an Omega E.Z.N.A.® Soil DNA Kit (D5625-02) and following the manufacturer’s instructions. A spectrophotometer was used to measure the concentration and purity of the isolated DNA (RS232G, Eppendorf, Germany).

#### 2.3.2. PCR amplification and sequencing

The primer pairs for AOB-*amoA*, AOA-*amoA*, comammox *Nitrospira* clade-A, and comammox *Nitrospira* clade-B were amoA1F/amoA2R, ArchamoAF /Arch-amoAR, comaA-244F /comaA-659R, and comaB-244F_a/comaB-659R_a, respectively (Wang J. et al., 2019; Jiang et al., 2020). PCR amplifications were performed in a total volume of 25 μl, containing 40 ng of DNA template, 1.0 μl of each primer (10 μM), 0.25 μl Q5 high-fidelity DNA polymerase, 5.0 μl 5× High GC Buffer, 5.0 μl 5× Reaction Buffer, and 0.5 μl deoxyribonucleoside triphosphate (dNTP, 10 mM). The amplifications were conducted with the following thermal conditions: 30 s initial denaturation at 98°C followed by 27 cycles of denaturation at 98°C for 15 s, annealing at 58°C/55°C/52°C/52°C (AOB/AOA/comammox clade-A/clade-B) for 30 s, extension at 72°C for 30/30/45/45 s (AOB/AOA/comammox clade-A/clade-B), and a final extension at 72°C for 7 min. The quantity and quality of PCR products were detected using a NanoDrop ND-1000 UV–Vis spectrophotometer. After successful amplifications, the PCR products were pooled in equimolar concentrations of 10 ng·μL^−1^ and sent to Personal Biotechnology Co., Ltd. (Shanghai, China) for paired-end 2 × 300 bp sequencing using the Illumina NovaSeq platform.

### 2.4. Data analysis

To identify significant differences between treatments, one-way analyses of variance (ANOVA) with Tukey’s HSD tests were carried out using SPSS 26.0. The richness and diversity indices (Chao1 and observed species; Simpson and Shannon) of AOA-amoA, AOB-amoA, comammox clade-A, and comammox clade-B were calculated using Mothur (version v.1.30.1). Redundancy analysis (RDA) was used to evaluate the connections between the diversity of the ammonia-oxidizing microbes and soil characteristics by the R ‘vegan’ package. Using the AMOS software (IBM SPSS AMOS 25), structural equation modelling (SEM) was used to evaluate the direct and indirect relationships between the diversity of the ammonia-oxidizing microbial community, agricultural management practices (such as fertilization, straw use, and earthworm), and soil physicochemical characteristics.

## 3. Results

### 3.1. Soil physicochemical properties

The impact of rice straw returning and earthworm addition on soil physicochemical properties was showed in Table 1. Compared with CK, the four fertilization treatments (RT, SR, W, and SRW) increased the SOC, AN, AP, NO_3_^−^-N, and NH_4_^+^-N contents and significantly decreased the soil pH. However, there was no significant difference between SR and RT treatments in each index (*p* > 0.05). Two earthworm addition treatments, W and SRW, significantly increased NH_4_^+^-N and AN contents (*p* < 0.05), and the AP and NO_3_^−^-N contents in SRW soil were also significantly higher than those of RT (*p* < 0.05).

**Table 1:** Soil physicochemical properties of rice straw returning and earthworm addition.

	pH	SOC %	TN %	TP %	AN mg·kg^−1^	AP mg·kg^−1^	NH_4_^+^-N mg·kg^−1^	NO_3_^−^-N mg·kg^−1^
CK	8.36 ± 0.06a	1.63 ± 0.09b	1.32 ± 0.05b	2.2 ± 0.1b	33.09 ± 1.51c	38.86 ± 2.11d	18.93 ± 1.01c	1.6 ± 0.07d
RT	8.21 ± 0.07b	2.45 ± 0.14a	1.45 ± 0.09ab	2.49 ± 0.12a	47.81 ± 3.1b	49.92 ± 2.01c	24.64 ± 1.11b	1.81 ± 0.12c
SR	8.25 ± 0.04b	2.38 ± 0.11a	1.53 ± 0.08a	2.39 ± 0.14ab	48.17 ± 2.41b	52.17 ± 2.79bc	25.24 ± 1.42b	1.87 ± 0.08bc
*W*	8.18 ± 0.04b	2.31 ± 0.17a	1.61 ± 0.11a	2.36 ± 0.06ab	55.66 ± 2.5a	55.57 ± 3.03ab	30.04 ± 1.57a	1.98 ± 0.1ab
SRW	8.16 ± 0.05b	2.54 ± 0.15a	1.62 ± 0.1a	2.31 ± 0.13ab	56.77 ± 2.78a	59.34 ± 2.84a	32 ± 1.46a	2.08 ± 0.04a

Dates in the table are Mean ± SE; Different letters in the same column indicate a significant difference (*p* < 0.05).

### 3.2. Alpha diversity of the ammonia-oxidizing microbial community

**Figure 1:**
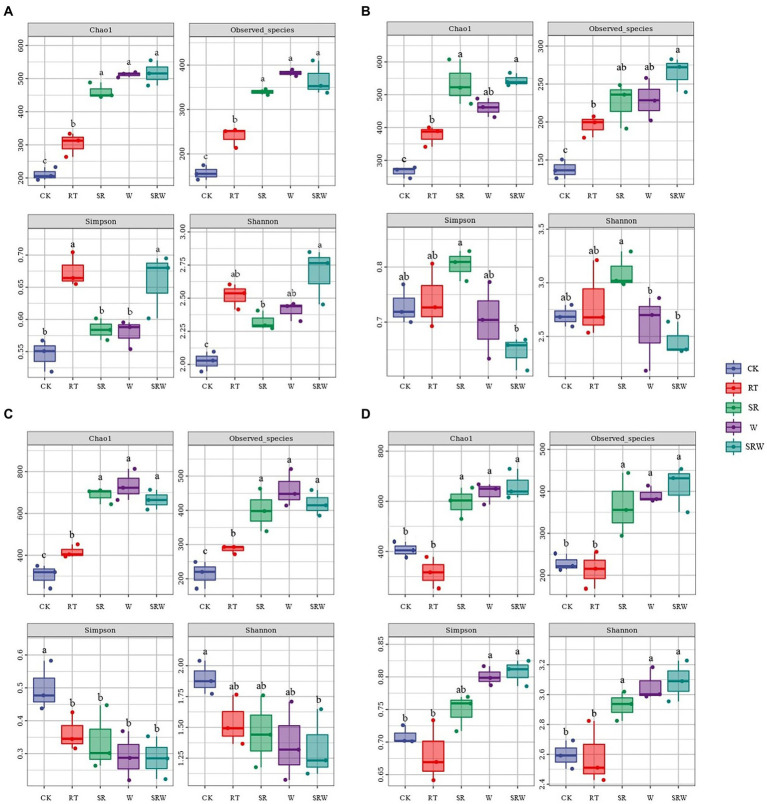
Richness and diversity indices of AOA **(A)**, AOB **(B)**, comammox clade-A **(C)**, and comammox clade-B **(D)**.

Chao1 and Observed species indexes were used as the microbial community richness indicators; Simpson and Shannon were employed to calculate the diversity. The results in Figure 1 showed that the ammonia-oxidizing microbial community diversities were significantly affected by earthworm addition and straw returning.

The richness of AOA, AOB, comammox clade-A, and comammox clade-B under all fertilization treatments was significantly higher than that under CK (*p* < 0.05), except for the richness of comammox clade-B under RT (*p* > 0.05). The richness of AOA, comammox clade-A, and comammox clade-B under W, SR, and SRW treatments was significantly higher than that under RT (*p* < 0.05). In SRW treatment, only the richness of AOB was notably higher than that under RT (p < 0.05).

Fertilization enhanced the AOA diversity, and the AOA diversity index value under SRW treatment was the highest. The SR treatment significantly increased the AOB diversity (p < 0.05), while SRW induced the lowest AOB diversity index. All the fertilization treatments decreased the comammox clade-A diversity, and there were no significant differences among them. The comammox clade-B diversity under W and SRW treatments was significantly higher than that under RT and CK treatments (p < 0.05).

### 3.3. Redundancy analysis of the ammonia-oxidizing microbial communities

**Figure 2:**
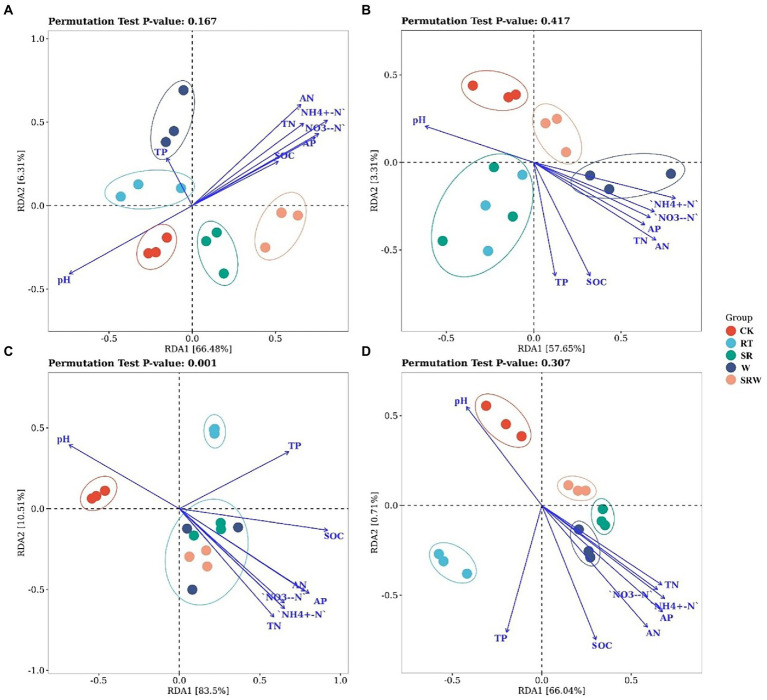
Redundancy analysis (RDA) of AOA **(A)**, AOB **(B)**, comammox clade-A **(C)**, and comammox clade-B **(D)** based on soil physicochemical properties.

The AOA community composition under straw returning treatments (SR and SRW) were separated from the other three treatments by RDA1, which explained 66.48% of the variation (Figure 2A). Moreover, the AOA composition under SR and SRW was positively correlated with the contents of NH_4_^+^-N (R^2^ = 0.783, *p* = 0.001), AN (R^2^ = 0.685, *p* = 0.002), and NO_3_^−^-N (R^2^ = 0.644, *p* = 0.005) and negatively correlated with pH (R^2^ = 0.602, *p* = 0.004). The AOB community composition under W and SRW treatments was separated from the other three treatments by RDA1, which explained 57.65% of the variation (Figure 2B). The AN (R^2^ = 0.556, *p* = 0.010), NH_4_^+^-N (R^2^ = 0.493, *p* = 0.015), and SOC (R^2^ = 0.471, *p* = 0.024) were positively correlated with the AOB composition under W and SRW treatments. The comammox clade-A community composition under all fertilization treatments (SR, RT, W, and SRW) was separated from CK by RDA1, which explained 83.50% of the variation. The SR, W, and SRW treatments were separated from RT treatment by RDA2, which explained 10.51% of the variation, but there was no significant separation between them (Figure 2C). The comammox clade-A composition under SR, W, and SRW was positively correlated with the contents of NO_3_^−^-N (R^2^ = 0.727, *p* = 0.001), NH_4_^+^-N (R^2^ = 0.767, *p* = 0.001), and AP (R^2^ = 0.887, *p* = 0.001) and negatively correlated with pH (R^2^ = 0.603, *p* = 0.006). The comammox clade-B community composition under SR, W, and SRW treatments was separated from CK and RT treatments by RDA1, which explained 66.04% of the variation (Figure 2D). The comammox clade-B community composition under SR, W, and SRW was positively correlated with AN (R^2^ = 0.648, *p* = 0.004), AP (R^2^ = 0.626, *p* = 0.005), and NH_4_^+^-N (R^2^ = 0.564, *p* = 0.006).

### 3.4. Potential nitrification rates and structural equation modelling analysis

**Figure 3:**
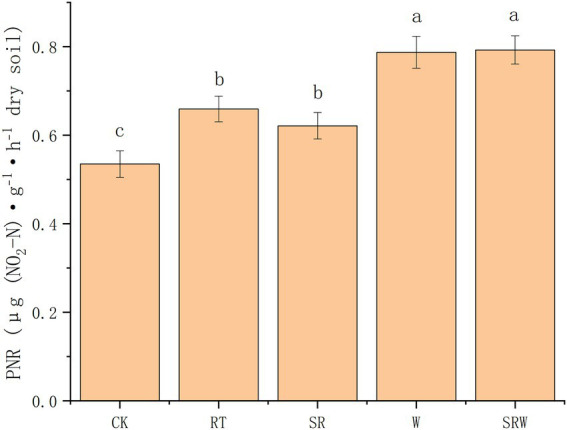
Potential nitrification rates (PNRs) under different agricultural management modes. Different letters indicate a significant difference (*p* < 0.05).

The soil PNRs under the five treatments were in the range of 0.535–0.793 μg N·g^−1^ dry soil·h^−1^ (Figure 3). The PNR under the fertilization treatments (SR, RT, W, and SRW) was significantly higher than that under CK treatment (*p* < 0.05). The PNR under W and SRW was significantly higher than that under RT and SR (*p* < 0.05), while there was no significant difference among RT and SR or W and SRW.

**Figure 4:**
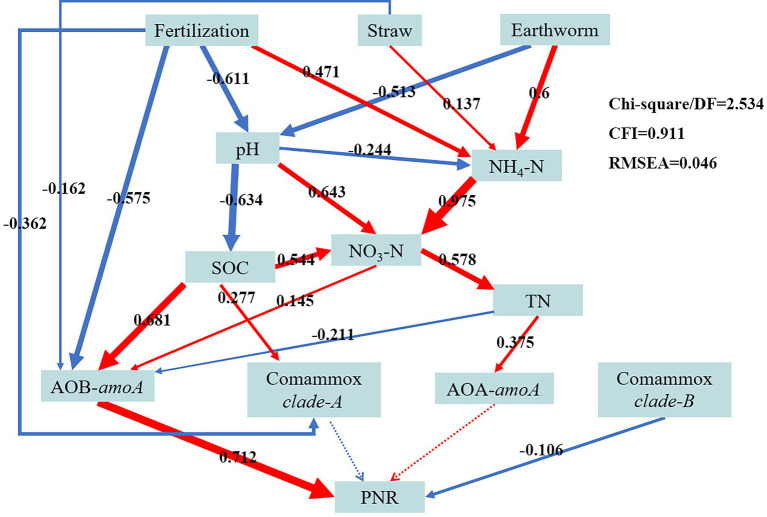
Structural equation modelling (SEM) showing the potential causal relationships among the AOA, AOB, comammox clade-A, and comammox clade-B abundances, agricultural management mode (including fertilization, straw, and earthworm), soil physicochemical factors (including pH, SOC, TN, NH_4_^+^-N, and NO_3_^−^-N), and potential nitrification rate (PNR). The arrow’s width indicates the intensity of the causal influence. The positive and negative correlations between the indicators are shown by the red and blue arrows, respectively. The path coefficients are represented by the numbers in the grey boxes above the arrows. Significant (*p* < 0.05) and insignificant (*p* > 0.05) routes are shown by solid and dotted lines, respectively.

To further explore the connections between the abundance of ammonia-oxidizing microorganisms, agricultural management practices (such as fertilization, straw use, and earthworm farming), and soil physicochemical variables, a structural equation model was created (Figure 4). NH_4_^+^-N was significantly affected by Fertilization (path coefficient = 0.471), Straw (path coefficient = 0.137), and Earthworm (path coefficient = 0.6). Fertilization (path coefficient = −0.611) and Earthworm (path coefficient = −0.513) both were significantly and negatively related to pH (alkaline soil). The SOC (path coefficient = 0.681) and NO_3_^−^-N (path coefficient = 0.145) were significantly and positively related to AOB microbes; while Fertilization (path coefficient = −0.575), Straw (path coefficient = −0.162), and TN (path coefficient = −0.211) were weakly related to AOB microbes. Comammox clade-A was positively related to Straw (path coefficient = −0.362) and negatively related to SOC (path coefficient = 0.277). And the TN (path coefficient = 0.375) was significantly and positively related to AOA microbes. The PNR was significantly positively correlated with AOB (path coefficient = 0.712), significantly negatively correlated with comammox clade-B (path coefficient = −0.106), and insignificantly correlated with neither AOB nor comammox clade-A.

## 4. Discussion

### 4.1. Effects of straw returning and earthworm addition on soil physicochemical properties

As an important agricultural management measure, straw returning can effectively enhance soil fertility and affect nutrients utilization in the soil (Yang et al., 2019). Earthworms are called “ecosystem engineers” (Shi et al., 2017) and can promote straw decomposition and accelerate soil nutrient cycling (Li et al., 2022). In our study, compared with RT treatment, SR did not significantly improve soil fertility (N and P contents; Table 1). This phenomenon might be due to the short duration (less than 2 years) of straw returning, resulting in little impact on the soil physicochemical properties (Cui et al., 2022). However, earthworm addition treatments (W and SRW) significantly increased the contents of available nutrients, such as AN, AP, NH_4_^+^-N, and NO_3_^−^-N (Table 1), which is in agreement with the results of Cheng et al. (2021) and Gong et al. (2018). Earthworm addition can improve the soil available nutrients owing to burrowing, casting, and feeding (Lemtiri et al., 2014). However, Ferlian et al. (2020) showed that earthworms decreased NH_4_^+^-N content and did not affect the NO_3_^−^-N content in a clay loam soil. Various soil types might be the reason for such difference (Lemtiri et al., 2014). Earthworms degrade clay loam aggregates through their natural activities, increasing soil porosity, and promoting mineral N leaching (Xu et al., 2018). Sandy loam soil was used in the current research, which had a rather loose soil texture and was less prone to earthworm infestation.

### 4.2. Effects of straw returning and earthworm addition on the alpha diversity of ammonia oxidizers

In previous studies, comammox microbes were more abundant than AOA and AOB and dominated in both acidic and alkaline soils (Wang J. et al., 2019). Comammox was 2.3 and 44.1 times more abundant than AOA and AOB, respectively, and the abundance of comammox tended to be higher in acidic soil (pH < 6.17; Hu et al., 2021). The results of the present research proved that, according to the alpha diversity, the ratio of AOA: AOB: clade A: clade B was 1: 1.08: 1.41: 1.33, the amount of comammox amoA genes were still dominant in the ammonia-oxidizing microbial community under alkaline soil conditions.

Our study showed that W, SR, and SRW treatments significantly increased the richness of the ammonia oxidizer community compared to CK (Figure 1). The richness of ammonia oxidizers was enhanced by earthworm addition. Wang et al. (2013) reported that the presence of earthworms intensified AOB diversity in soil. Furthermore, Xue et al. (2022) concluded that earthworms increased the gene (*amoA*) abundance of AOB and AOA by improving soil structure and providing C sources. Ammonia oxidizers were indirectly affected by the improved soil aeration and oxygenation caused by earthworm burrowing and casting activities (Xu et al., 2018). Straw returning similarly increased the richness of the ammonia oxidizer community. Straw returning increased the organic carbon and nitrogen in the soil (Jin et al., 2020), which directly provided a large amount of nutrients to ammonia oxidizers, thus promoting their growth.

In the present study, SRW treatment increased the AOA community diversity, while W and SR did not, since earthworms can promote straw decomposition (Elyamine and Hu, 2020). A great quantity of organic N was increased in the soil through straw returning, while available N was released by the feeding and casting activities of earthworms (Edwards and Arancon, 2022). According to some previous studies, AOA is an oligotrophic microorganism with a high affinity for substrates (NH_4_^+^-N; Jung et al., 2021; Liu and Yang, 2021), and its response to increasing substrates is not significant (Taylor et al., 2012). Additionally, Zhalnina et al. (2012) suggested that AOA growth and activity appeared to be encouraged by high NH_4_^+^-N concentrations. Tourna et al. (2011) found that AOA thrived on medium with NH_4_^+^-N concentrations as high as 15 mM, but experienced growth inhibition at 20 mM. This inhibitory concentration was much higher than that previously reported (2–3 mM; Hatzenpichler et al., 2008; Martens-Habbena et al., 2009). This inconsistency suggested that the response mechanism of AOA to different concentrations of ammonium in soil may be influenced by various factors (such as soil texture or pH). The AOB community diversity indices were the highest under SR treatment and were the lowest under SRW treatment. Straw returning increased the AOB diversity, which is consistent with the earlier research findings (Zhang et al., 2019). The straw overlaid on the soil was beneficial for the ability to retain water and nutrients, increase the sustainability of nutrients delivery (Siedt et al., 2021), which could supply AOB with a large number of nutrients. However, the AOB diversity was decreased by SRW treatment in the present study (Figure 1); this result might be ascribed to the substantially increased NH_4_^+^-N and NO_3_^−^-N concentrations by SRW treatment compared to SR (Table 1). As opposed to the whole AOB taxon, several specific hosts of AOB amoA were more abundant by the comparatively high NO_3_^−^-N and NH_4_^+^-N contents (Lv et al., 2020). Fertilization reduced the comammox clade-A community diversity; the reason might be that comammox clade-A can survive in a low-nitrogen niche and excessive nutrients might reduce its diversity (Hu and He, 2017). Comammox clade-B can also grow actively and autotrophically in paddy fields (Wang J. et al., 2019). In this study, both straw returning and earthworm addition improved the comammox clade-B community diversity. He et al. (2022) suggested that NO_3_^−^-N was a key factor affecting the comammox clade-B community, which had a higher NO_3_^−^-N tolerance. In the present study, W and SRW treatments significantly increased the NO_3_^−^-N concentration (*p* < 0.05; Table 1), stimulating the growth of the comammox clade-B community.

### 4.3. Response of ammonia oxidants to soil physicochemical properties

Studies have suggested that the AOB community structure was affected by different fertilizers applied and that chemical N fertilizer stimulates AOB growth more than organic fertilizer (Ai et al., 2013); (Zou et al., 2022). The AOB community was altered by chemical N fertilizer and earthworm addition (Figure 2). The mineralization of soil N was accelerated, and the NH_4_^+^-N content in the soil was increased, through the burrowing, feeding, and casting activities of earthworms (Lemtiri et al., 2014). The impact of chemical N fertilizer on the AOA community structure was less significant (Strauss et al., 2014). However, the AOA community under the two straw returning treatments was significantly separated from that of the other treatments. This indicates that rice straw was added to soil as a kind of organic matter, resulting in an increased C/N, which affected the AOA community composition (Dai et al., 2021). Genome analysis showed that low-affinity Rh-type ammonium transporters existed merely in the genome of clade-A, while high-affinity AmtB-type transporters were found in clade-B (Palomo et al., 2016). This explained why the comammox clade-B community was more sensitive to straw and earthworms than that of clade-A.

Soil pH is a vital driver of the ammonia oxidizer community composition (Prosser and Nicol, 2012; Stempfhuber et al., 2015). Soil pH directly or indirectly controls the ammonia oxidizer community because it may affect the content and availability of matrix and microorganism growth and activity (Nicol et al., 2008). Studies have suggested that AOA and comammox clade-A could be well adapted to acidic soils (Tripathi et al., 2015); (Shi et al., 2018), while AOB and comammox clade-B were found to grow in alkaline soils (Jiang et al., 2015). In our study, pH (pH = 8.16 ~ 8.36) was negatively correlated with the AOA and comammox clade-A community compositions but not with the AOB and comammox clade-B community compositions. Soil NH_4_^+^-N acted as a substrate for ammonia oxidizers and thus influenced their community compositions (Li et al., 2020). A previously report showed that AOA was more abundant in soils with lower AN levels, while AOB became more abundant in soils with higher AN levels (Prosser and Nicol, 2012). The comammox community structure was significantly correlated with the N addition amount, while NO_3_^−^-N and AN played key roles in influencing the comammox community structure (Wang J. et al., 2019; Wang Z. et al., 2021). In our study, all ammonia oxidant community compositions were positively correlated with NH_4_^+^-N; this is consistent with the results of Gao et al. (2016). However, the most important factor crucial to the AOB and comammox clade-B community structures was AN, while that to the clade-A community structure was NO_3_^−^-N.

### 4.4. Relative contributions of ammonia oxidants to nitrification

The present study showed that the treatments with earthworm addition (W and SRW) significantly increased PNR, while SR treatment did not, compared to RT treatment. The PNR under W and SRW was 1.19 and 1.20 times that under RT, respectively. Earthworm addition significantly increased the agricultural soil PNR. This finding in the present study supports some recent reports which suggested that earthworm addition enhanced soil PNR in agricultural soil (Xu et al., 2018); (Chen et al., 2014). Earthworm addition increased soil aeration through burrowing and other natural activities, thereby increasing NH_4_^+^-N and PNR, which was beneficial for nitrification (Xu et al., 2018). The critical environmental factors affecting the soil PNR were soil pH and NH_4_^+^-N. The change in pH affected other soil physicochemical properties (SOC; NH_4_^+^-N; NO_3_^−^-N), which indirectly influenced the PNR. NH_4_^+^-N, as the reaction substrate of the ammonia oxidation process, also affected the PNR.

Ammonia oxidants are crucial to the nitrification process, and it is debatable to what extent comammox, AOA, and AOB contribute to nitrification. Zhang et al. (2019) and Wang J. et al., (2017) reported that nitrification was driven by AOB rather than AOA in alkaline and nitrogen-rich agricultural soils. This is basically consistent with our result that AOB dominated the nitrification process. Interestingly, comammox clade-B was negatively correlated with the PNR (path coefficient = −0.106, *p* < 0.05), whereas comammox clade-A was not (*p* > 0.05). This might be because comammox clade-B was grown in alkaline soils, whereas comammox clade-A grew well in acidic soils (Tripathi et al., 2015); (Shi et al., 2018); (Jiang et al., 2015). Comammox clade-B used the same substrate (NH_4_^+^-N) as AOB for the ammonia oxidation process, and there was a certain overlap between their niches.

## 5. Conclusion

In this study, Illumina high-throughput sequencing was used to assess how ammonia oxidizers responded to 2-year of sequential straw returning and earthworm addition in an alkaline paddy soil. The results showed that earthworm addition was more conducive to improving soil physicochemical properties and PNR than straw returning, and the effect of straw returning combined with earthworm addition was the most beneficial method. Compared to RT treatment, W and SRW treatments significantly increased the contents of soil available nutrients (*p* < 0.05), and the PNR under W and SRW treatments increased by 1.19 and 1.20 times, respectively. The richness indices of the ammonia oxidizer community were enhanced by straw returning and earthworm addition. The environmental driving factors of the ammonia oxidation community compositions were soil pH, NH_4_^+^-N, and NO_3_^−^-N. Under nitrogen-rich and alkaline conditions, AOB microbes played dominant roles in soil nitrification. Straw returning combined with earthworm addition in paddy field directly and indirectly increased soil nutrient content, and improved soil nitrification by changing soil ammonia oxidizers community structure. In subsequent studies, more accurate techniques, such as ^13^C-DNA stable isotope probing (DNA-SIP), should be applied to determine the nitrification activities and contributions of ammonia oxidizer microorganisms.

## Date availability statement

The datasets presented in this study can be found in online repositories. The names of the repository/repositories and accession number(s) can be found at: NCBI, PRJNA868943 (https://www.ncbi.nlm.nih.gov/bioproject/PRJNA868943).

## Author contributions

All authors listed have made a substantial, direct, and intellectual contribution to the work and approved it for publication.

## Funding

This study was supported by Agriculture Research System of Shanghai, China (Grant No. 202303), Shanghai; Agriculture Applied Technology Development Program, China (T20180414); the Outstanding Team Program of Shanghai Academy of Agricultural Science [Grant no. Hu-Nong-Ke-Zhuo 2022 (008)].

## Conflict of interest

The authors declare that the research was conducted in the absence of any commercial or financial relationships that could be construed as a potential conflict of interest.

## Publisher’s note

All claims expressed in this article are solely those of the authors and do not necessarily represent those of their affiliated organizations, or those of the publisher, the editors and the reviewers. Any product that may be evaluated in this article, or claim that may be made by its manufacturer, is not guaranteed or endorsed by the publisher.
